# Targeting Gut Microbiota, Inflammation, and Metabolic Dysfunction-Associated Steatotic Liver Disease in Type 2 Diabetes Mellitus: Therapeutic Strategies and the Emerging Role of Time-Restricted Feeding

**DOI:** 10.3390/cells15191831

**Published:** 2026-10-08

**Authors:** Sthembiso Msane, Andile Khathi

**Affiliations:** Department of Human Physiology, School of Medicine, College of Health Sciences, University of KwaZulu-Natal, Durban 4000, South Africa; 219023719@stu.ukzn.ac.za

**Keywords:** gut microbiota, prediabetes, time-restricted feeding, inflammatory response, liver, metabolism

## Abstract

Time-restricted feeding has emerged as a promising non-pharmacological intervention for the management of metabolic disorders, particularly type 2 diabetes mellitus and its associated complications. Time-restricted feeding involves restricting food intake to a specific daily eating window while fasting for the remaining hours within 24 h. Furthermore, time-restricted feeding can be combined with specific dietary modifications to enhance its therapeutic benefits. Type 2 diabetes mellitus has been strongly linked to gut dysbiosis, adipose tissue dysfunction, chronic low-grade inflammation, metabolic dysfunction-associated steatotic liver disease, and an increased risk of acute pancreatitis. Given the rising global prevalence of type 2 diabetes mellitus, early intervention strategies are becoming increasingly important. Prediabetes, as an intermediate stage preceding type 2 diabetes mellitus, provides a critical opportunity to understand the mechanisms underlying disease progression and to develop effective preventive strategies. However, the effects of time-restricted feeding on gut dysbiosis, adipose tissue dysfunction, dysregulated inflammatory responses, metabolic dysfunction-associated steatotic liver disease and the risk of acute pancreatitis at prediabetic state remain incompletely understood. Therefore, this review summarizes the current evidence regarding the therapeutic potential of time-restricted feeding in the management of type 2 diabetes mellitus and its related complications, while highlighting areas that require further investigation.

## 1. Introduction

The microorganisms living in the intestinal tract affect human health and nutritional processes through various pathways [1,2]. Recent studies indicate that the gut microbiota is closely linked to host metabolism through the production of microbial metabolites [3,4]. These include short-chain fatty acids (SCFAs), which provide energy to colonic epithelial cells, trimethylamine-N-oxide (TMAO), which is associated with the development of white adipose tissue, and inosine-5-monophosphate (IMP), which plays a role in lipid metabolism [3,4,5]. Furthermore, the gut microbiota can influence the host’s immune system and regulate bile acid composition, thereby impacting digestion and overall metabolism [6,7]. The connection between gut microbiota and the onset of diabetes and other metabolic disorders in the host may stem from toxic metabolites produced by the microbiota, increased energy extraction from food by these microbes, and low-grade inflammation associated with endotoxemia [8,9].

Adipose tissue serves not only as a significant source of inflammatory markers but also as a target of inflammation in diabetes [10]. An inflammatory state has also been observed in various other organs involved in regulating metabolic homeostasis, including the liver, hypothalamus, skeletal muscle, and the pancreas [11,12,13,14]. This mechanism seems to depend strongly on interleukin-1 (IL-1), leading some researchers to propose that type 2 diabetes mellitus may include an autoinflammatory aspect similar to that seen in type 1 diabetes mellitus [15]. Acute pancreatitis (AP) is characterized by a complex series of events [16]. A key early step is the premature activation of pancreatic zymogens within the cells, leading to autodigestion of the pancreas [16]. Normally, the pancreas employs several protective mechanisms to prevent intracellular zymogen activation and self-digestion. However, in pancreatitis, these defenses become impaired or are overwhelmed [17].

In the liver, homeostatic inflammatory processes regulate hemodynamic alterations, capillary permeability, leukocyte infiltration into tissues, and the release of inflammatory mediators [18]. However, in conditions such as Metabolic Dysfunction-Associated Steatotic Liver Disease (MASLD) and Metabolic Dysfunction-Associated steatohepatitis (MASH), a leading cause of impaired liver function in Western populations, these processes become dysregulated and are frequently associated with obesity and diabetes [19,20,21,22]. In obese individuals, excess abdominal fat disrupts lipid and glucose metabolism, leading to fat accumulation in the liver, which contributes to insulin resistance [23,24]. This insulin resistance is closely linked to chronic, low-grade inflammation, driven by a range of mediators released from immune cells and adipocytes [19,23].

With the growing shift away from drug-based treatments toward non-pharmacological approaches, intermittent fasting (IF) has emerged as a promising alternative strategy [25]. IF refers to a range of eating patterns in which individuals cycle between extended periods of normal calorie consumption and periods of little to no energy intake [26]. Recent findings have emphasized the application of IF and its effectiveness in managing metabolic-related disorders [27,28]. IF can take several forms, including the 5:2 diet, alternate-day fasting, and time-restricted feeding [25,29,30]. Time-restricted feeding (TRF) is a type of intermittent fasting that involves a designated period of fasting each day, during which individuals abstain from food for specific hours and consume meals within the remaining hours of a 24 h cycle [31,32]. Terminology depends on the study model: time-restricted eating (TRE) is used in human studies, whereas TRF is the preferred term in animal studies [31,32,33].

The TRF regimen typically includes various fasting-to-eating schedules such as 16:8, 14:10, 12:12, 20:4, and 18:6 h [26,32]. Additionally, both early and late TRF approaches have been implemented to assess the effectiveness and potential benefits of this dietary strategy [34,35]. TRF can be implemented alongside specific dietary modifications to enhance its overall effectiveness [36]. Research has indicated that calorie restriction on its own may be less effective than when it is combined with an additional intervention strategy [36,37,38]. Previous research on glucose metabolism has shown that a 14:10 h TRF pattern can help regulate body weight and improve glucose control in prediabetes [31]. This review aims to highlight the potential role of a TRF regimen as a non-pharmacological strategy for the prevention and management of prediabetes and type 2 diabetes mellitus. To provide scientific rationale for this approach, the review first examines the pathophysiological mechanisms underlying prediabetes, including gut dysbiosis, adipose tissue dysfunction, chronic low-grade inflammation, MASH, and pancreatitis. It then reviews the benefits and limitations of current pharmacological therapies before discussing the available evidence supporting TRF as a complementary dietary intervention. Finally, the review identifies the limited evidence regarding the effects of a 14:10 h TRF regimen in the prediabetic state and highlights key areas for future mechanistic research.

### Methodology Section

This narrative review was undertaken to synthesize current evidence on the relationships among type 2 diabetes mellitus, prediabetes, gut microbiota, inflammation, MASLD)/MASH, and the therapeutic roles of antidiabetic agents, including metformin, sulfonylureas, dipeptidyl peptidase-4 inhibitors (DPP-4i), and sodium–glucose cotransporter-2 inhibitors (SGLT2i), as well as the emerging potential of IF, particularly TRF.

A comprehensive literature search was conducted using PubMed, Scopus, Web of Science, and Google Scholar to identify relevant peer-reviewed publications published between January 2010 and June 2026. Seminal studies published before 2010 were also included where appropriate to provide historical context and to explain key biological mechanisms underpinning the pathophysiology of type 2 diabetes mellitus and its associated metabolic complications.

The search strategy combined Medical Subject Headings (MeSH) and free-text keywords, including “type 2 diabetes mellitus,” “prediabetes,” “gut microbiota,” “gut dysbiosis,” “intermittent fasting,” “time-restricted feeding,” “metabolic dysfunction-associated steatotic liver disease,” “MASLD,” “MASH,” “non-alcoholic steatohepatitis,” “acute pancreatitis,” “chronic inflammation,” “intestinal permeability,” “lipopolysaccharide,” “metformin,” “sulfonylurea,” “SGLT2 inhibitors,” “DPP-4 inhibitors,” and “short-chain fatty acids.” Boolean operators (AND, OR, and, where appropriate, NOT) were used to combine search terms and refine the search strategy.

Eligible studies included original research articles, randomized controlled trials, prospective and retrospective cohort studies, systematic reviews, meta-analyses, case reports, and high-quality experimental animal studies published in English. Studies were included if they investigated the pathophysiological relationships among type 2 diabetes mellitus, prediabetes, gut microbiota, inflammatory responses, MASLD/MASH, and pancreatitis, or evaluated the metabolic effects of intermittent fasting, time-restricted feeding, metformin, sulfonylureas, DPP-4 inhibitors, or SGLT2 inhibitors. Conference abstracts, editorials, duplicate publications, and studies lacking relevance to the objectives of this review were excluded.

The retrieved evidence was synthesized narratively due to heterogeneity in study designs, interventions, and reported outcomes as shown in Figure 1. Throughout the review, evidence is categorized according to study type (human clinical and animal experiments) to distinguish the strength and translational relevance of the available evidence and to facilitate critical interpretation of the findings.

## 2. Metabolic Disorders Related to Prediabetes

### 2.1. The Role of Gut Microbiota in the Development of Metabolic Disorders

The human gastrointestinal (GI) tract hosts a vast and diverse microbial community comprising over 100 trillion microorganisms [1,39]. In the colon, bacterial density is estimated to range from 10^11^ to 10^12^ cells per milliliter, making it one of the most densely populated microbial environments on Earth [40]. The gut microbiome contains more than 3 million genes that generate thousands of metabolites, in contrast to the human genome, which has approximately 23,000 genes [40]. The gut microbiota is made up of both advantageous and potentially harmful microbes in the digestive system that collectively contribute to proper bodily functioning [2]. The main groups of microbes in the gut include Firmicutes, Bacteroidetes, Actinobacteria, Proteobacteria, Fusobacteria, and Verrucomicrobia [41]. Among these, Firmicutes and Bacteroidetes make up about 90% of the gut microbiota [2,41,42]. An imbalance in the gut microbiota (dysbiosis) is believed to contribute to various diseases in the host, including obesity, type 2 diabetes mellitus, cancer, and inflammatory bowel disorders (IBDs) [43].

When blood glucose levels increase, insulin is released to facilitate the entry of glucose into the cells, acting as the primary regulator of glucose homeostasis [44]. However, insulin is not the sole factor involved in this process, as the gut microbiota also plays an important role in regulating glucose levels, as summarised in Figure 2 [45,46]. A balanced gut microbiota helps preserve the integrity of intestinal tight junctions and the gut barrier, preventing harmful substances like lipopolysaccharides (LPS) from entering the bloodstream [47,48]. If this barrier becomes compromised, systemic inflammation can arise, impairing insulin signaling and disrupting glucose control [48]. Persistent, low-grade systemic inflammation is widely regarded as a major factor driving insulin resistance, metabolic syndrome, and type 2 diabetes mellitus [49,50].

Metabolic conditions such as obesity and prediabetes are strongly influenced by the amount and distribution of body fat, as differences in adiposity affect both fatty acid storage capacity and adipokine secretion [51]. Adipose tissue is a type of connective tissue that stores energy as fat [52]. When functioning properly, it plays an important role in maintaining metabolic balance [53]. However, when it becomes dysfunctional, it can promote chronic inflammation and contribute to metabolic diseases such as prediabetes [51]. Furthermore, a study reported that the intestinal bacteria most commonly altered in both mild acute pancreatitis (MAP) and severe acute pancreatitis (SAP) patients were closely linked to inflammation, suggesting a role for the gut microbiota in the progression of acute pancreatitis [54].

### 2.2. Chronic Low-Grade Inflammation Contributes to Glucose Dysregulation

AP is among the most common acute abdominal conditions, with a rising incidence [54]. Although most cases are mild and resolve on their own, SAP is associated with high morbidity and mortality, primarily due to early multiple organ failure and the subsequent development of infectious complications [54,55]. SAP is characterized by necrosis of the pancreatic tissue and adjacent structures, accompanied by an intense inflammatory response involving the infiltration of neutrophils and macrophages [56]. The pathophysiology of AP is complex and mainly involves the early activation of digestive enzymes within the pancreas, resulting in autodigestion and a subsequent inflammatory cascade Figure 2 [57]. This process starts with the inappropriate conversion of trypsinogen to trypsin inside the acinar cells instead of the duodenum [58]. Triggers such as ductal blockage, disturbances in calcium balance, and alterations in pH can initiate this premature enzyme activation [17,59].

According to the Atlanta classification, AP is broadly divided into two forms: interstitial edematous AP, which involves inflammation of the pancreatic tissue and surrounding areas, and necrotizing acute pancreatitis, which is characterized by necrosis of the pancreatic parenchyma and peripancreatic tissues [60]. Mutations in genes such as cystic fibrosis transmembrane conductance regulator (CFTR), protease serine 1/cationic trypsinogen (PRSS1), chymotrypsin C (CTRC), and serine protease inhibitor Kazal type 1 (SPINK1), which play roles in the activation and regulation of trypsinogen, have been linked to recurrent acute pancreatitis and its progression to chronic pancreatitis [60]. Trypsinogen, a serine protease, is believed to be the first enzyme activated, which then triggers the activation of other digestive proenzymes [17,59]. Notably, visceral adipose tissue (VAT) volume is significantly associated with the development of pancreatic pseudocysts and systemic inflammatory response syndrome in patients with AP, and increased VAT levels may contribute to the progression toward SAP [61]. Additionally, increased visceral adiposity has been identified as a more accurate predictor of severe pancreatitis compared with body mass index (BMI) [62].

Adipose tissue functions as an endocrine organ by secreting hormones and signaling molecules that help control metabolism, appetite, and inflammatory processes [10]. It consists of white adipose tissue (WAT), the predominant form, and brown adipose tissue, which appears to play a minimal role in inflammatory processes [14]. WAT is further divided into subcutaneous and visceral (abdominal) fat depots [14]. Abdominal WAT plays a key role in inflammatory processes, functioning as a source of cytokines and a wide range of bioactive molecules involved in inflammation, such as tumour necrosis factor (TNF)-α, interleukins (IL) including IL-1, IL-6, and IL-10, as well as leptin, adiponectin, monocyte chemoattractant protein, resistin, angiotensinogen, visfatin, retinol-binding protein-4, and serum amyloid protein, among others [10,14,63]. Adipose tissue dysfunction also plays a causal role in the development of MAFLD, as it promotes the buildup of intrahepatic triglycerides (IHTGs) [51,64,65]. This occurs through enhanced lipid delivery to the liver, driven by insulin resistance and the release of pro-inflammatory adipokines [64,65,66].

Adipose tissue pathophysiology is characterized by elevated levels of inflammatory cytokines, reflecting ongoing chronic low-grade inflammation [51,65]. Inflammation is a component of the body’s innate immune system and is a vital process that not only protects against harmful bacteria and pathogens but also contributes to tissue maintenance and repair [67]. Although metabolic dysfunction, insulin resistance, and tissue injury can initiate or amplify pro-inflammatory activity in macrophages, the resulting inflammation further promotes insulin resistance and subsequent hyperinsulinemia [67,68]. Hyperinsulinemia is defined by abnormally high levels of insulin in the bloodstream relative to glucose levels, and it usually does not cause low blood glucose [69,70]. When the body’s cells become less responsive to insulin, the pancreas increases insulin production to compensate. Over time, this results in chronically elevated insulin levels [71]. Persistently high insulin promotes fat storage by stimulating lipogenesis and inhibiting lipolysis, which contributes to increased fat accumulation, particularly in the abdominal area and hyperglycemia Figure 2 [72,73].

### 2.3. Chronic Hyperglycemia Contributes to MASLD

Formerly known as non-alcoholic fatty liver disease (NAFLD), MASLD is one of the most prevalent chronic liver diseases worldwide, affecting more than 30% of the global population [74]. MASLD is a range of liver conditions characterized by the accumulation of large fat droplets in liver cells, occurring in people who do not consume alcohol in amounts that would damage the liver [22,75]. The likelihood of developing MASLD increases with higher body weight and the presence of type 2 diabetes mellitus [75]. A proportion of cases of MASLD can progress to more severe stages, including MASH, fibrosis, cirrhosis, and ultimately liver failure [76,77]. Formerly known as non-alcoholic steatohepatitis (NASH), MASH is the more aggressive subtype of MASLD and may replace hepatitis C as the leading cause of liver transplantation [78,79,80]. The metabolic imbalance that results in excessive fat accumulation in the liver is thought to arise from three main sources: (i) increased delivery of dietary fats to the liver from the gut, due either to higher intake or altered gut function and microbiome; (ii) greater release of free fatty acids (FFAs) from the non-esterified pool, primarily originating from white adipose tissue; and (iii) enhanced de novo fat synthesis in the liver driven by excess carbohydrate intake and hyperinsulinemia associated with adipose tissue insulin resistance [23,24,81]. When these processes are not balanced by an increased secretion of very low-density lipoprotein (VLDL) from the liver or utilization through other metabolic pathways, triglycerides and free fatty acids accumulate in hepatocytes [82]. The relationship between MASLD and Type 2 diabetes is both strong and multifaceted, with MASLD occurring at a notably high prevalence among individuals with type 2 diabetes mellitus [83,84]. Type 2 diabetes mellitus is preceded by a transitional metabolic state known as prediabetes [85,86].

### 2.4. Prediabetes

Prediabetes is a condition in which blood glucose levels are higher than normal but not high enough to meet the criteria for diabetes [87]. This condition is associated with an increased risk of progressing to type 2 diabetes mellitus [87]. The World Health Organisation (WHO) does not recognize HbA1c as a diagnostic criterion for prediabetes [88]. In contrast, the American Diabetes Association (ADA) classifies individuals as prediabetic if they have impaired fasting glucose (IFG) of 5.6–6.9 mmol/L, impaired glucose tolerance (IGT) of 7.8–11.0 mmol/L, or a glycated haemoglobin (HbA1c) level of 5.7–6.4% [89]. IGT is typically associated with insulin resistance in skeletal muscle and reduced glucose uptake, whereas IFG is primarily linked to hepatic insulin resistance and increased glucose production by the liver [88,89]. Gut microbiota play a key role in regulating inflammation, and disruptions in microbial balance (dysbiosis) can promote both local and systemic inflammatory responses [90].

Increased intestinal permeability may allow bacterial products such as LPS to enter circulation, triggering immune activation and contributing to chronic low-grade inflammation [90]. This inflammatory state is implicated in the development and progression of NASH, where it drives liver injury, fat accumulation, and fibrosis [91,92]. Similarly, in AP, gut-derived inflammatory mediators and microbial translocation can exacerbate pancreatic inflammation and tissue damage [93,94].

### 2.5. Gut–Liver–Adipose Tissue–Pancreas Axis

Importantly, these metabolic disturbances are increasingly recognized as interconnected processes within a broader metabolic-inflammatory network, rather than as isolated pathological events [95,96,97,98]. The key pathophysiological events include: (I) gut microbiota dysbiosis and increased intestinal permeability, promoting the translocation of bacterial LPS and FFAs to the liver; (II) activation of inflammatory pathways, including Toll-like receptor 4 (TLR4)/nuclear factor kappa B (NF-κB) contributing to hepatic insulin resistance and systemic inflammation; (III) adipose tissue dysfunction and increased lipolysis associated with insulin resistance; (IV) hepatic lipid accumulation due to metabolic dysregulation; and (V) pancreatic β-cell compensation followed by functional decline under prolonged glucolipotoxic stress [47,48,50,60,64,82,95,96,97,98]. Collectively, these interconnected mechanisms contribute to the development and progression of gut dysbiosis and MASLD/MASH, while potentially increasing vulnerability to pancreatic complications, including acute pancreatitis [95,96,97,98]. Therefore, targeting the gut–liver–pancreas axis, metabolic pathways, and inflammatory processes may provide a more comprehensive strategy for managing these interconnected metabolic disorders. Regardless of the diagnostic criteria currently used to define prediabetes, the coexistence of insulin resistance, inflammation, gut microbiota dysregulation, MASH, and either impaired fasting glucose, impaired glucose tolerance, or both continues to confer a risk of progression to type 2 diabetes mellitus [83,99,100,101,102,103,104]. Different strategies have been implemented to reduce the prevalence of prediabetes and its progression to type 2 diabetes mellitus and its related complications [25].

## 3. Lifestyle Modification and Pharmacological Management of Prediabetes

The standard approach towards the management of prediabetes includes lifestyle modifications such as dietary intervention and increased physical activity, often complemented by pharmacological interventions, particularly metformin [105,106]. Research studies indicate that, compared with metformin, lifestyle modification is more effective in reducing the risk of developing type 2 diabetes mellitus [105,107]. Guidelines from the ADA also recommend that individuals with prediabetes adopt lifestyle modifications to prevent the onset of type 2 diabetes mellitus, including maintaining a healthy diet, increasing physical activity, and achieving weight loss [108]. Conversely, the anti-diabetic drug market has shown consistent growth in volume, with newly approved medications [108]. Several factors, including efficacy, safety, and cost considerations, influence the selection of oral anti-diabetic drugs for patients with type 2 diabetes mellitus [108,109,110,111]. However, dietary quality remains a fundamental determinant of gut microbiota composition, intestinal barrier integrity, and systemic inflammation, all of which contribute to the pathogenesis and progression of type 2 diabetes mellitus [112,113]. High dietary quality is achieved through adherence to a balanced and healthy dietary pattern characterized by a high intake of fruits, vegetables, high-fiber whole grains, and fish, together with the consumption of low-fat dairy products, lean protein sources, and foods low in added sugars [114,115].

Adherence to such a dietary pattern has been associated with a more favorable gut microbiota composition, which may promote intestinal barrier integrity, enhance the production of SCFAs, and attenuate systemic low-grade inflammation [115]. Furthermore, evidence from the National Health and Nutrition Examination Survey (NHANES) 2007–2018 suggests that a higher Dietary Index for Gut Microbiota (DI-GM) score is associated with a lower risk of cardiovascular disease (CVD), supporting the potential role of dietary intervention strategies aimed at modulating the gut microbiota [116]. Similarly, evidence from an observational study suggests that sustained adherence to a high-quality diet, particularly when combined with leisure-time physical activity, is associated with a lower risk of type 2 diabetes mellitus among urban Chinese adults [117]. Although dietary quality is an important component of the prevention and management of type 2 diabetes mellitus, pharmacological therapy remains necessary for many individuals [116,118]. Furthermore, evidence suggests that treatment adherence and persistence may differ according to the prescribed class of antidiabetic medication [119]. Notably, antidiabetic medications may be used either as monotherapy or in combination with lifestyle modifications [118].

### 3.1. Metformin

Metformin is an antidiabetic drug classified within the biguanide class [120]. Metformin is the most widely used medication for managing patients with type 2 diabetes mellitus [121]. It controls glucose production via both adenosine monophosphate-activated protein kinase (AMPK)-dependent and independent mechanisms [122]. Nonetheless, accumulating evidence indicates that its main site of action is the gastrointestinal tract [122]. Recent studies in diet-induced obese mice and clinical studies involving patients with type 2 diabetes mellitus suggest that this intervention may modify the gut microbiota by promoting the growth of the mucin-degrading bacterium Akkermansia muciniphila and several SCFA-producing bacterial species [123,124,125]. Prolonged metformin use in patients with type 2 diabetes mellitus has been associated with an increased risk of vitamin B12 deficiency, which may contribute to the development of peripheral neuropathy [126]. Clinical evidence suggests that IBD is associated with lower vitamin B12 levels, with patients diagnosed with IBD being more likely to exhibit vitamin B12 deficiency than healthy individuals [127,128].

IBDs are chronic immune-mediated conditions marked by recurrent flare-ups that lead to ongoing damage of the intestinal barrier and disruption of the gut microbiota balance [127]. Evidence from a *Drosophila* model suggests that vitamin B12 may promote gut repair following injury by reducing excessive cell proliferation and inflammatory activity. However, the clinical relevance of these findings remains to be established in human studies [127]. Clinical evidence also suggests that reduced folate and vitamin B12 levels are associated with elevated homocysteine concentrations, an independent risk factor for cardiovascular disease, particularly in individuals with type 2 diabetes mellitus [126,129]. Notably, metformin concentrations in the gut are 100 to 300 times higher than in the bloodstream, making the GI tract the main site of metformin accumulation in humans [123,130]. Beyond its established effects on improving insulin sensitivity and suppressing hepatic gluconeogenesis, mechanistic and preclinical studies suggest that metformin may modulate inflammation, oxidative stress, and cellular metabolism, particularly in adipose tissue [131]. A nationwide retrospective cohort study conducted in Taiwan reported that metformin use was associated with a lower risk of pancreatitis than the use of sulfonylureas or DPP-4 inhibitors [132].

Acute pancreatitis caused by metformin is extremely uncommon and typically occurs as a result of both drug overdose and impaired kidney function [133]. Evidence from a randomized controlled trial suggests that metformin has limited effects on liver function tests and liver histology in non-diabetic individuals with insulin resistance and MASH [134]. Alterations in the intestinal microbiota may contribute to some of metformin’s glucose-lowering effects [135]. However, they may also lead to adverse outcomes, such as gut dysbiosis and gastrointestinal symptoms, which can negatively affect patient tolerance and adherence to metformin [135].

#### 3.1.1. Metformin Effects on the Gut Microbiota

Long-term use of metformin for four years or more has been associated with a 41.0% increased risk of vitamin B12 deficiency [136]. In addition, each additional year of use is linked to a further 5% increase in the risk of developing B12 deficiency [136]. Conversely, studies have explored the use of metformin in combination with lifestyle modifications [137,138,139,140]. Notably, lifestyle modifications and dietary interventions, including probiotic supplementation, may help correct gut microbiome dysbiosis and support the management of type 2 diabetes mellitus [141]. Clinical trial evidence suggests that a multi-strain probiotic may reduce the incidence of gastrointestinal side effects in patients with type 2 diabetes mellitus who are intolerant to metformin [142]. Although long-term metformin use has been associated with vitamin B12 deficiency, mechanistic and clinical evidence suggests that modulation of the gut microbiota, particularly through an increased abundance of Akkermansia species, may contribute to the antidiabetic effects of metformin, providing a potential explanation for its therapeutic efficacy in patients with type 2 diabetes mellitus [123]. This also suggests that targeting the gut microbiota to enhance Akkermansia abundance may represent a potential therapeutic strategy for type 2 diabetes mellitus [123]. Clinical evidence from a study involving women with type 2 diabetes mellitus suggests that metformin use may be associated with an increased abundance of pro-inflammatory bacteria and a reduced abundance of SCFA-producing bacteria [143]. A preclinical study in a rat model of MASLD suggested that early intervention with Lactobacillus reuteri and metronidazole (MTZ) in combination with metformin may attenuate the progression of MASLD and liver injury by modulating gut dysbiosis, inflammatory processes, and autophagic pathways [144].

#### 3.1.2. Metformin Effects on Liver Function

Although the precise mechanism of metformin remains incompletely understood, the liver is regarded as one of its main target organs [145]. Metformin is known to lower hepatic glucose production, but this alone does not fully account for its effects, with growing evidence suggesting that the gut also plays an important role in its overall action [123,127,146]. Additionally, long-term metformin use has been associated with a reduced risk of all-cause mortality, liver transplantation, and hepatocellular carcinoma in diabetic patients with MASH and advanced fibrosis [147]. Interestingly, a study suggested that, as the prevalence of type 2 diabetes mellitus and related metabolic disorders continues to increase in the United States, the use of metformin is also expected to rise [148]. This may, in turn, lead to a higher occurrence of rare idiosyncratic liver toxicity, emphasizing the importance of clinical vigilance for this potential adverse effect [148,149]. On the other hand, studies recommend combining metformin therapy with dietary interventions to improve overall treatment outcomes [150,151,152,153]. A study suggested that metformin is not effective as a preventive strategy for MASLD [154]. However, it may be beneficial in its treatment when used alongside dietary intervention [151,153]. Nevertheless, preclinical evidence from LSL-KrasG12D/+; p48-Cre (KC) mice suggests that metformin may attenuate high-fat, high-carbohydrate diet (HFCD)-induced weight gain, hepatic steatosis, hyperleptinemia, and hyperinsulinemia. The study also reported improvements in metabolic profiles and a reduction in the tumour-promoting effects of diet-induced obesity on pancreatic ductal adenocarcinoma (PDAC) development [154].

#### 3.1.3. Metformin Effects on the Pancreas

A rare adverse effect of metformin is acute pancreatitis, which has been reported in cases of overdose or impaired renal function [155,156]. The absence of common causes of acute pancreatitis, including gallstones, alcohol consumption, hypercalcaemia, hypertriglyceridaemia, and trauma, further supported the diagnosis [155]. Furthermore, two case reports linked drug-induced renal failure, precipitated by angiotensin-converting enzyme (ACE) inhibitors, angiotensin receptor blockers (ARBs), non-steroidal anti-inflammatory drugs (NSAIDs), including Ibuprofen and Celecoxib, as well as diuretics, with impaired metformin excretion, which led to drug toxicity, severe lactic acidosis, and pancreatitis [133,157]. Mechanistic evidence suggests that metformin may exacerbate both acute and chronic pancreatitis, potentially through increased release of oxidized mitochondrial DNA (Ox-mtDNA) via modulation of mitochondrial iron homeostasis and NINJ1-mediated plasma membrane rupture, whereas lifestyle modification is recommended for the management of acute pancreatitis [158,159]. Acute pancreatitis was generally believed to originate from early pathological events within pancreatic acinar cells [160,161]. Early acinar cell injury triggered a localized inflammatory response, which, when severe, progressed to systemic inflammatory response syndrome (SIRS) [162]. Excessive SIRS may subsequently contribute to distant organ injury and multiple organ dysfunction syndrome (MODS), the principal contributor to morbidity and mortality associated with acute pancreatitis [162]. Recent studies suggest that inflammatory mediators play a significant role in the pathogenesis of acute pancreatitis and the development of MODS [161]. Acute pancreatitis can be associated with severe clinical consequences [59]. Therefore, preventing or reducing the risk of recurrence and progression to chronic pancreatitis, which is associated with an elevated risk of pancreatic cancer, requires proactive and, in some patients, long-term management [59].

### 3.2. Sulfonylureas

Sulfonylureas, the earliest class of oral antidiabetic medications, continue to be among the most frequently prescribed treatments for type 2 diabetes mellitus, alongside metformin [163,164]. Gliclazide and glibenclamide are commonly used sulfonylurea antidiabetic drugs [165,166]. Sulfonylureas are advised as a supplementary therapy to metformin in most international guidelines and can be combined with all classes of oral antidiabetic drugs except for glinides [167]. The use of sulfonylureas as a monotherapy has declined over time and is primarily recommended for patients who are unable to take metformin [168]. Current clinical evidence suggests that gliclazide has minimal effects on the gut microbiota, with one recent study reporting no significant changes in gut microbial composition following 12 weeks of treatment in patients with diabetes [169,170]. Nevertheless, findings from a recent cross-sectional study suggest that this relationship may not be bidirectional and that gut microbiota dysbiosis may be associated with a diminished therapeutic response to sulfonylureas [171]. Among patients with type 2 diabetes mellitus on metformin, initiation of empagliflozin was not associated with an increased risk of acute pancreatitis compared to initiation of sulfonylurea therapy [172].

One clinical study reported that sulfonylurea therapy in patients with non-insulin-dependent diabetes mellitus (NIDDM) was associated with improved insulin-stimulated peripheral glucose uptake, potentially through enhanced insulin-mediated glucose transport and lipogenesis in adipose tissue, together with increased glycogen synthase activity in skeletal muscle [173]. Evidence from a preclinical study suggests that this treatment exerts antidiabetic effects in primary human adipocytes and obese mouse models [174]. Mechanistic evidence from preclinical studies indicates that modulation of PPARγ phosphorylation may contribute to the extra-pancreatic effects of sulfonylureas on adipocytes in both in vitro and in vivo models, rather than traditional agonistic activity at clinically relevant doses [174]. Sulfonylurea use is independently associated with a higher risk of major cardiovascular events [175]. Relative to sulfonylureas, treatment with SGLT2 inhibitors, thiazolidinediones, and DPP-4 inhibitors was associated with an increased probability of MASLD regression [176]. Sulfonylureas are generally well tolerated; however, their long-term use can lead to side effects, including hypoglycemia and weight gain [177].

#### 3.2.1. Effects of Sulfonylureas on Gut Modulation

Evidence from preclinical studies suggests that gliclazide may attenuate acetic acid-induced colonic injury in rats by modulating inflammatory responses, including reducing pro-inflammatory mediators and increasing anti-inflammatory cytokines [167]. Its protective effects are associated with upregulation of PPARγ, downregulation of NF-κB, and inhibition of MAPK and caspase-3 activity [167]. Furthermore, sulfonylureas have been reported to exert beneficial modulatory effects in IBD [178]. Interestingly, a recent clinical study reported that 12 weeks of gliclazide treatment did not result in significant changes in the gut microbiota composition of patients with type 2 diabetes mellitus, suggesting that any metabolic effects of gliclazide are unlikely to be primarily mediated through alterations in the colonic microbiota [170]. Conversely, Evidence from a preclinical study suggests that glibenclamide may modestly influence gut microbiota alpha diversity in diabetic rats, with an increased abundance of members of the Paraprevotellaceae family and Prevotella species being observed [166]. Notably, a preclinical study reported that co-administration of gliclazide with mono-keto-cholic (MKC) acid reduced the bioavailability of MKC in healthy rats receiving probiotics, whereas no significant effect was observed in probiotic-treated rats with type 1 diabetes mellitus [179]. These findings suggest that probiotics may influence MKC absorption or metabolism in healthy animals, while no comparable effects were observed in diabetic rats [179].

#### 3.2.2. Effects of Sulfonylureas on Liver Function

Managing diabetes mellitus with sulfonylureas in patients with cirrhosis has been shown to be complex because these agents undergo hepatic metabolism [180]. While most clinical guidelines regard sulfonylurea as safe for use, some recommend reducing the dosage [180,181]. Emerging evidence from observational clinical studies suggests that sulfonylurea therapy in patients with type 2 diabetes mellitus may be associated with progressive deterioration of liver function [182,183]. However, evidence from a preclinical study suggests that glibenclamide may exert hepatoprotective effects in high-fat diet (HFD)-fed rats, with observed reductions in blood glucose, triglyceride and cholesterol levels, deoxyribonucleic acid (DNA) damage, apoptosis, and inflammatory markers, alongside enhanced antioxidant defenses and improved insulin signaling pathways [184]. Notably, mechanistic evidence from a preclinical study indicates that alpha-lipoic acid (ALA) was associated with reduced gliclazide-induced liver toxicity in type 2 diabetic rats, with the observed effects potentially involving suppression of inflammation and apoptosis together with enhancement of hepatic antioxidant pathways [185]. It has been reported that, compared with metformin, DPP-4 inhibitors, SGLT-2 inhibitors, and combination therapies, sulfonylureas are strongly associated with significantly elevated liver function markers such as Alanine aminotransferase (ALT), aspartate aminotransferase (AST), and alkaline phosphatase (ALP), and bilirubin, whereas the other therapies show similar hepatic profiles [186]. The use of sulfonylureas in patients with type 2 diabetes mellitus and cirrhosis is complex because these agents undergo hepatic metabolism, and impaired liver function may influence their safety and therapeutic profile [180,181]. Although some studies suggest hepatoprotective effects of certain sulfonylureas and protective adjunct therapies such as alpha-lipoic acid, clinical evidence also associates these agents with elevated liver function markers compared with other antidiabetic therapies [184,185]. Overall, this implies that sulfonylurea therapy in patients with liver disease should be used cautiously, with careful dose adjustment and monitoring of hepatic function.

#### 3.2.3. Effects of Sulfonylureas on Pancreas

The association between type 2 diabetes mellitus and acute pancreatitis is multi-faceted and influenced by a range of interrelated factors, including the underlying disease process, associated comorbidities and pharmacological therapies [187]. Clinical studies have reported that long-term sulfonylurea therapy may improve pancreatic islet function in patients with NIDDM [188]. It is suggested that the primary glucose-lowering effect of sulfonylureas is largely driven by this action on the pancreas [188]. Emerging observational evidence suggests potential associations between glibenclamide use, obesity, and an increased risk of acute pancreatitis. Furthermore, obesity has been associated with prolonged hospital stays in patients with acute pancreatitis [189]. Furthermore, a recent observational study suggested that sulfonylurea use may be associated with a higher risk of pancreatitis than treatment with DPP-4 inhibitors or metformin [132]. Contrary to this, Evidence from another preclinical study suggests that glyburide treatment may attenuate pancreatic injury in genetically obese ob/ob mice with SAP, as reflected by reduced relative pancreatic mass, pancreatic water content, and cellular death compared with vehicle-treated obese SAP mice [190]. Collectively, evidence regarding the relationship between sulfonylurea therapy and pancreatitis remains inconsistent, with findings varying across clinical and preclinical studies [188,189,190]. While some observational studies suggest a potential association between sulfonylurea use and pancreatitis risk, experimental models have reported possible protective effects under specific conditions.

### 3.3. DPP4 Inhibitors

Over recent years, DPP-4 inhibitors have increasingly supplanted sulfonylureas as the preferred second-line therapy following metformin failure, and numerous fixed-dose combinations of metformin and DPP-4 inhibitors are currently available [191]. DPP-4 inhibitor therapy should be discontinued when initiating Glucagon-like peptide-1 (GLP-1) receptor agonists [191]. However, DPP-4 inhibitors can be administered as monotherapy in patients for whom metformin is contraindicated or not tolerated [191]. DPP-4 inhibitors have been reported to influence gut microbiota composition, which may contribute to their glucose-lowering effects and represent a potential additional therapeutic mechanism [192,193]. Additionally, altering microbial composition and the resulting functional shifts may offer a promising approach to improving glucose regulation [192,193]. Research findings also indicate that DPP-4 inhibition is associated with extra-pancreatic protective effects, including reduced diet-induced adipose tissue inflammation and hepatic steatosis in diabetic mice [194].

Relative to DPP-4 inhibitors, GLP-1 receptor agonists, pioglitazone, and sulfonylureas, SGLT-2 inhibitor therapy was associated with a lower risk of cirrhosis, liver failure, and all-cause mortality among patients with type 2 diabetes mellitus [195]. A recent study was consistent with phase III clinical trial findings, reporting that gastrointestinal and skin reactions are among the most commonly reported adverse drug reactions (ADRs) across the DPP-4 inhibitor class [196]. These reactions are believed to arise from potential drug interactions, primarily through effects on DPP-4 activity and/or structure homolog (DASH) proteins, which amplify the immune-inflammatory modulation of DPP-4 [196]. There is limited evidence supporting the combined use of DPP-4 inhibitors and lifestyle modification.

#### 3.3.1. Effects of DPP-4 Inhibitors on the Gut

The association between DPP4 and the gut microbiome is important for developing DPP-4-targeted therapies aimed at maintaining microbiome balance and regulating immune responses in IBD [197]. A study reported that DPP4, derived from both host and microbial sources and present in membrane-bound and soluble forms, is associated with intestinal fibrosis in Crohn’s disease (CD) [198]. Preclinical findings suggest that DPP4 may represent a potential therapeutic target, and dual-source inhibition may offer a promising approach for limiting stricture formation in CD [198]. Nevertheless, further experimental and clinical studies are required to determine the efficacy and safety of this strategy [198]. DPP4 expression, activity, and substrate levels have been shown to vary in response to different stimuli, including artificial sweeteners [199]. Vildagliptin has been reported to modulate gut microbiota composition, which may influence hepatic immune responses [193].

#### 3.3.2. Effects of DPP-4 Inhibitors on the Liver

Hepatopulmonary syndrome (HPS), characterized by arterial hypoxemia, occurs in association with liver diseases such as cholestasis [200]. Preclinical rat models, including common bile duct ligation (CBDL), have been used to replicate key features of HPS and investigate its underlying mechanisms [200]. Evidence from a preclinical rat model of liver transplantation suggests that hepatoprotective strategies may have potential therapeutic benefits in HPS. Given the involvement of DPP-4 in multiple pathways associated with liver disease, targeting this molecule may represent a potential therapeutic approach that warrants further investigation [200].

In this context, a preclinical study reported that prophylactic vildagliptin improved liver fibrosis, hypoxemia, and survival in CBDL rats, potentially through hepatoprotective mechanisms involving antioxidant, anti-inflammatory, anti-angiogenic, and anti-fibrotic effects, as well as attenuation of pathological intrahepatic vasodilation [200]. In vivo, improvement of MASH was associated with reduced TRAIL receptor-mediated apoptosis and decreased hepatic DPP4 expression [201]. Similarly, in vitro studies have shown that DPP-4 inhibitor treatment reduces markers of TRAIL-mediated lipo-apoptosis and downregulates DPP-4 expression in palmitate-exposed hepatocytes [201]. Overall, preclinical findings suggest that DPP4 inhibition may attenuate amylin liver NASH (AMLN) diet-induced MASH in mice by reducing lipotoxicity-associated apoptosis, potentially through modulation of hepatic DPP4 expression [201]. In addition, evidence from preclinical studies suggests that sitagliptin may protect intestinal epithelial cells by attenuating oxidative stress and inflammatory responses in vitro and in a mouse model of SAP [202].

#### 3.3.3. Effects of DPP-4 Inhibitors on the Pancreas

The estimated risk of pancreatitis with DPP-4 inhibitors appears reassuring, but wide confidence intervals include both possible harm and benefit [203]. Overall, the data support a likely lack of increased pancreatitis risk, though uncertainty remains [203]. A preclinical study reported that vildagliptin enhanced pancreatic β-cell survival in type 2 diabetic rats, potentially through improved glycaemic control and reductions in β-cell apoptosis and oxidative stress [204]. Analysis of the United States Food and Drug Administration Adverse Event Reporting System has identified reports of acute pancreatitis among patients receiving DPP-4 inhibitors, including cases of severe necrotizing and haemorrhagic pancreatitis [205]. These pharmacovigilance signals suggest a potential safety concern; however, they do not confirm a causal relationship due to limitations inherent to spontaneous adverse event reporting [205]. While the large nationwide cohort study conducted in Taiwan demonstrated that the use of DPP4 inhibitors was not significantly associated with an increased risk of acute pancreatitis, even among diabetic patients considered high risk, these findings further reinforce the safety profile of incretin-based therapies in vulnerable populations [206].

The mechanism of action of DPP-4 inhibitors remains incompletely defined. However, their principal effect is attributed to the inhibition of endogenous GLP-1 degradation, resulting in a modest increase in physiological GLP-1 concentrations [207]. Given that this rise in GLP-1 is both transient and limited in magnitude, it is unlikely to fully account for the clinically relevant glucose-lowering effects observed with DPP4 inhibition when considered in isolation [207]. Accordingly, these observations suggest that additional or complementary mechanisms may contribute to the therapeutic effects of these agents beyond the enhancement of GLP-1 receptor signalling [207]. Prolonged GLP-1 receptor activation in the exocrine pancreas has been proposed as a potential mechanism that may influence pancreatic exocrine function, including changes in secretion dynamics [208].

### 3.4. SGLT2 Inhibitors

SGLTs are sodium–glucose transporters located on the luminal membrane of the proximal tubule, where they reabsorb approximately 180 g (1 mol) of glucose daily from the glomerular filtrate [209]. SGLT2 inhibition promotes urinary glucose and calorie excretion, leading to reductions in both plasma glucose levels and body weight [210]. The SGLT2 inhibitor class includes agents such as sergliflozin, remogliflozin, ipragliflozin, dapagliflozin, and empagliflozin [211,212]. A preclinical study reported that dapagliflozin treatment improved arterial stiffness, endothelial function, and vascular smooth muscle function in type 2 diabetic mice, while also inducing modest alterations in gut microbiota composition [213]. These vascular benefits were associated with decreases in hyperglycemia and circulating inflammatory markers [213]. SGLT2 inhibition decreases glucose transporter (GLUT) 4 gene and protein expression in abdominal subcutaneous adipose tissue, suggesting a shift in substrate utilization away from glucose oxidation and lipid storage, likely through reduced glycerol production [214,215].

The selective SGLT2 inhibitor ipragliflozin has been reported to ameliorate hyperglycaemia and MASH-related features in type 2 diabetic mice, suggesting that ipragliflozin, either alone or in combination with metformin, may warrant further investigation as a potential therapeutic strategy for managing type 2 diabetes mellitus with MASH [216,217]. Empagliflozin monotherapy has been reported to attenuate MASH-related features through anti-steatotic and anti-inflammatory effects, while combination therapy with linagliptin has been associated with greater improvements in MASH outcomes, including enhanced anti-fibrotic effects [218]. Mild genital mycotic infections represent the most frequently reported adverse effect of SGLT2 inhibitors, whereas the incidence of urinary tract infections is only marginally elevated [219]. Infrequent adverse events include volume depletion-related complications, euglycemic diabetic ketoacidosis, and, in the case of canagliflozin, a heightened risk of bone fractures and lower limb amputations [219]. However, these risks have not been consistently reported in other trials or with other SGLT2 inhibitors [219]. Overall, serious adverse events are uncommon and typically arise under particular predisposing conditions [219].

#### 3.4.1. Effects of SGLT2-Inhibitors on the Gut

Preclinical studies suggest that empagliflozin may attenuate atherosclerosis partly through modulation of the gut microbiota, with some of these beneficial effects being transferable through microbiota transplantation experiments [220]. Similarly, another preclinical study reported that dapagliflozin treatment increased the abundance of beneficial bacterial taxa in the intestine of a type 2 diabetic rat model [221]. Overall, evidence from animal studies suggests that SGLT2 inhibitors, including empagliflozin, dapagliflozin, and canagliflozin, are associated with alterations in gut microbiota composition, including enrichment of SCFA-producing bacteria such as Bifidobacterium and Bacteroides and reductions in potentially pathogenic genera such as Clostridium and Shigella [222,223]. These microbial shifts are associated with higher butyrate levels, lower LPS concentrations, and improved inflammatory profiles [222,223]. On the other hand, in patients with type 2 diabetes mellitus, clinical studies have reported that the combination of dapagliflozin with Bifidobacterium probiotics improves metabolic parameters and is associated with favorable changes in gut microbiota composition [224]. Notably, correlation analysis suggested that alterations in the microbiota were positively associated with liver fibrosis and glucose metabolism [225].

#### 3.4.2. Effects of SGLT2-Inhibitors on the Liver

SGLT2 inhibitors may represent a promising novel therapeutic option for the treatment of MASLD in patients with type 2 diabetes mellitus [226]. Preclinical studies indicate that SGLT2 inhibitors may reduce hepatic steatosis, improve insulin sensitivity, and attenuate liver inflammation and fibrosis in experimental models [227,228]. Clinical studies suggest that SGLT2 inhibitor use in patients with MASLD may improve liver-related outcomes, including reductions in serum markers of liver injury and potential improvements in liver function [229,230,231]. However, a case report described elevated liver function biomarkers in a patient receiving therapy, with liver biopsy findings consistent with drug-induced liver injury [232]. This report highlights the need for increased awareness of the potential hepatotoxic effects of dapagliflozin, especially in individuals with pre-existing chronic liver disease [232]. Rare case reports have described cholestatic drug-induced liver injury in patients receiving empagliflozin, including individuals with cirrhosis [233]. In contrast, canagliflozin treatment has been reported to reduce serum glucose, HbA1c, triglyceride, uric acid, and ferritin levels, as well as FIB-4 index scores and body weight, while improving liver function parameters [234]. SGLT2 inhibitors may be beneficial for patients with MASLD and type 2 diabetes because they improve liver fat accumulation, insulin resistance, inflammation, and overall metabolic and liver function [227,228]. However, there are rare reports of drug-induced liver injury with some agents in this class, particularly in patients with underlying liver disease, indicating potential safety concerns [233]. On the other hand, another study demonstrated that dapagliflozin may be a promising therapeutic option for MASLD, as it effectively reduces hepatic and pancreatic fat accumulation while improving glycaemic control [235].

#### 3.4.3. Effects of SGLT2-Inhibitors on the Pancreas

SGLT-2 inhibitors may be associated with an increased risk of acute pancreatitis, particularly within the first six months of treatment [236,237,238]. This risk appears to be significantly higher when combined with DPP-4 inhibitors, GLP-1 receptor agonists, or ACE inhibitors [236]. Additionally, the specific type of SGLT-2 inhibitor and its combination with statins have been identified as factors that may influence the risk of mortality following acute pancreatitis [236]. However, an observational study using data from two large United States databases evaluated the risk of acute pancreatitis associated with empagliflozin in patients with type 2 diabetes mellitus [176]. The study reported that, among metformin-treated patients with type 2 diabetes mellitus, initiation of empagliflozin was not associated with an increased risk of acute pancreatitis compared with initiation of sulfonylureas [176]. Furthermore, although the exact prevalence of drug-induced pancreatitis is difficult to determine due to the reliance on case reports, it appears to be a rare adverse event associated with SGLT2 inhibitors, including empagliflozin [239,240]. However, several case studies reported a very rare but serious adverse effect, acute pancreatitis, associated with canagliflozin use [241,242,243].

A case study reported that a patient had started dapagliflozin five days before admission, in addition to long-term treatment with insulin detemir, sitagliptin, metformin, and rosuvastatin [244]. Symptoms resolved after stopping sitagliptin and dapagliflozin [244]. However, when dapagliflozin was reintroduced one year later due to worsening glycaemic control, the patient developed a recurrent episode of acute pancreatitis, suggesting a potential causal relationship as reported in Table 1 [244]. Several potential mechanisms have been proposed to explain drug-induced acute pancreatitis, including pancreatic duct constriction, metabolic disturbances, cytotoxic effects, accumulation of toxic metabolites or intermediaries, idiosyncratic reactions, and hypersensitivity responses [245,246,247]. Early recognition of drug-induced pancreatitis may facilitate appropriate clinical management and potentially reduce complications among affected individuals [242].

## 4. Intermittent Fasting

Given the adverse effects linked to pharmacological therapies used for the treatment and management of type 2 diabetes mellitus and prediabetes, there is an increasing demand for alternative therapeutic approaches associated with minimal or less severe side effects [127,128,206,220]. Compared with pharmacological therapies, the adverse effects reported with IF are generally less severe and are often transient [176,248]. Potential contraindications or circumstances requiring caution with IF/TRF include individuals with low body weight or those at increased risk of malnutrition, children and adolescents, older adults, and women who are pregnant or breastfeeding [249,250,251]. When TRF is combined with glucose-lowering therapy, closer monitoring of blood glucose levels may be necessary to assess the effects of the combined intervention and minimize the risk of hypoglycaemic events [251,252,253,254]. Reported side effects include disrupted social eating patterns, hunger, irritability, palpitations, dizziness, headache, abdominal pain, mood changes, vomiting, and, in some individuals, hypoglycaemia [176,248,254,255]. These effects are typically manageable and may diminish as individuals adapt to the fasting regimen, although tolerability can vary depending on the duration of fasting, dietary composition, and individual metabolic status [176,248,254,255]. IF has attracted increasing research attention as a potential alternative intervention for the treatment and management of type 2 diabetes mellitus [255,256,257,258]. IF protocols encompass dietary strategies characterized by alternating periods of eating with fasting phases, during which energy intake is either completely restricted or significantly reduced to minimal or zero caloric consumption [25,258]. The timing of fasting and feeding periods differs across IF protocols, including approaches such as the 5:2 diet, alternate-day fasting, and TRF [25,259].

The TRF regimen generally comprises several fasting-to-feeding schedules, including 16:8, 14:10, 12:12, 20:4, and 18:6 h fasting-to-eating ratios [26,32]. These ratios represent the number of fasting hours followed by the number of hours allocated for food intake within 24 h. For example, a 16:8 schedule involves fasting for 16 consecutive hours and consuming all daily meals within an 8 h window, whereas a 14:10 or 12:12 schedule provides a longer eating period and may be more suitable for beginners [260,261]. More intensive approaches, such as 18:6 or 20:4, extend the fasting duration and shorten the eating window, potentially enhancing metabolic adaptations but requiring greater adherence and planning [26,260]. Additionally, both early and late TRF approaches have been employed to evaluate the efficacy and potential benefits of this dietary intervention [34,35].

TRF can be implemented alongside specific dietary modifications to enhance its overall effectiveness [36]. Individuals may combine TRF with calorie control, macronutrient adjustments such as higher protein or lower carbohydrate intake, or adherence to specific dietary patterns [36,262]. Integrating TRF with balanced, nutrient-dense food choices may further support metabolic health, weight management, and overall well-being [36]. Research suggests that calorie restriction alone may provide fewer substantial benefits compared with approaches that combine caloric reduction with additional intervention strategies [36,37,38]. Furthermore, a recent preclinical study investigating glucose metabolism reported that a 14:10 h TRF regimen was associated with reductions in body weight and improvements in glycaemic control in prediabetic male Sprague–Dawley rats [31]. Several preclinical studies have demonstrated that TRF may improve multiple features associated with metabolic syndrome in both male and female mice, including reductions in body weight, adiposity, glucose intolerance, plasma cholesterol levels, and plasma triglyceride concentrations, alongside improvements in cardiac function [263,264,265].

### 4.1. The Contribution of TRF to Changes in Gut Microbiota

Regarding its effects on the gut microbiota, several studies have reported that TRF is associated with alterations in microbial composition and relative abundance, as shown in Figure 3 [266,267,268,269]. By promoting microbial abundance and diversity, TRF may influence host metabolism and contribute to improved nutritional status [266,267,268,269]. Dietary composition significantly influences the structure and function of the gut microbiota [270,271]. Clinical studies suggest that low-carbohydrate and low-protein dietary approaches may improve glucose tolerance and modulate gut microbiota composition in individuals with type 2 diabetes mellitus [270,271]. Preclinical evidence from eight-week-old male Kunming mice suggests that TRF during high-fat diet exposure may modulate gut microbial composition and regulate circadian patterns of hepatic lipid metabolism-related proteins [272]. Moreover, preclinical evidence from aged rat models suggests that TRF combined with dietary intervention may induce significant changes in gut microbiota composition [273]. Restricting food access to the same daily time enables TRF to elicit food-anticipatory activity driven by the endogenous circadian rhythm [274]. Evidence from another study suggests that childhood TRF may have detrimental effects, leading to irreversible metabolic abnormalities, reduced immune competence, and impaired pubertal progression [275]. Overall, current evidence suggests that TRF can influence gut microbial composition and host metabolic regulation, with outcomes likely dependent on factors such as dietary composition, age, and the timing of food intake [270,271,274]. However, the long-term implications of TRF-induced microbial changes remain to be fully established.

### 4.2. The Role of TRF in the Progression of MASH

TRF has also been shown to reduce the expression of genes associated with hepatic lipogenesis and inflammation while increasing the expression of genes involved in fatty acid β-oxidation [276]. Consequently, preclinical evidence from diet-induced obese mice suggests that TRF may improve insulin sensitivity and attenuate markers of liver injury [276]. Additionally, preclinical evidence from obese mouse models of MASLD suggests that short-term isocaloric TRF may reduce hepatic inflammation independently of major changes in body weight or hepatic steatosis [277]. This suggests that the duration of fasting is an important determinant of the degree of improvement, as TRF may improve liver metabolism and insulin function through gene regulation, reduce inflammation even in the short term, and may require a longer intervention period to reduce hepatic fat accumulation effectively [276,277]. In mouse models of diet-induced MASH, TRF has been associated with improvements in hepatic pathology and alterations in Per2-related signalling pathways [278]. TRF may serve as a complementary approach for managing diabetes and MASH, while potentially slowing MASH progression in individuals with type 2 diabetes mellitus [279]. It has been reported that, although the sustained effects of TRF may differ across tissues and genes, its influence on adipose tissue inflammation and immune cell infiltration can persist for several weeks, which may partly support the maintenance of insulin sensitivity even after cessation of TRF [280]. Overall, the available preclinical evidence suggests that TRF may improve liver health by enhancing hepatic metabolism, increasing insulin sensitivity, reducing inflammation, and modulating the expression of genes involved in lipid metabolism and circadian regulation [276,277,279]. These findings support the potential of TRF as a complementary dietary strategy for attenuating MASLD/MASH progression and improving metabolic outcomes in individuals with type 2 diabetes mellitus.

### 4.3. The Contribution of TRF to the Regulation of Adipose Tissue Dysfunction and Inflammation

Furthermore, preclinical evidence from male C57BL/6 mice suggests that TRF may mitigate obesity-associated changes in immune cell composition and is associated with improved insulin sensitivity [281]. TRF can also activate thermogenesis in brown adipose tissue and reduce inflammatory markers, leading to improvements in hepatic steatosis and decreased accumulation of white adipose tissue [282]. A preclinical study demonstrated that TRF increased uncoupling protein 1 (UCP1) expression in WAT and was associated with reduced age-related whitening of brown adipose tissue in aged mice [283]. Moreover, TRF has been reported to enhance mitochondrial metabolic activity within WAT depots [283]. It also reduced macrophage infiltration, promoted a beneficial shift in macrophage (M) polarization by lowering the M1/M2 ratio, and attenuated adipose tissue fibrosis [283]. Another study reported that TRF protected lean and obese mice against weight gain, normalized the expression of inflammatory and fibrotic genes, and reduced adipose tissue collagen deposition and hepatic triglyceride accumulation induced by light exposure, either alone or in combination with obesity [284]. A TRF protocol has been demonstrated to support weight management in the context of a high-fat diet, with earlier daytime eating conferring enhanced metabolic benefits [31,285]. However, the elevated levels of inflammatory markers observed in the TRF groups indicate that dietary composition may still promote inflammation, even in the absence of apparent obesity [285]. Collectively, these findings highlight the potential of TRF to modulate adipose tissue inflammation, immune responses, and mitochondrial function in the type 2 diabetes mellitus state.

### 4.4. The Potential Role of TRF in Modulating Acute Pancreatitis

As previously noted, increased visceral adiposity has been identified as a stronger and more accurate predictor of severe pancreatitis than BMI [61]. Furthermore, non-fasting mild-to-moderate hypertriglyceridemia (≥2 mmol/L) is linked to an increased risk of acute pancreatitis, with higher hazard ratios than those observed for myocardial infarction [286]. Evidence from a randomized clinical trial suggests that both a low-calorie diet and an 8 h TRE intervention, either alone or in combination, may reduce body weight and subcutaneous fat in humans [33]. However, TRE showed greater benefits than LCD in improving visceral obesity and cardiometabolic health outcomes [33]. There are different TRE regimens based on the timing of the eating window, including early TRE (eTRE), which involves consuming food earlier in the day, and late TRE (lTRE), which involves skipping breakfast [287]. In high-fat diet-fed mouse models, early TRF has been associated with improvements in metabolic and hepatic parameters compared with late TRF and unrestricted feeding, including reduced body weight, adipose tissue accumulation, circulating glucose, insulin, cholesterol, leptin, TNF-α, and ALT levels [35]. Overall, TRF under a high-fat diet, whether implemented early or late, was associated with decreased inflammation and reduced fat accumulation relative to AL-HF-fed mice [35]. Clinical evidence indicates that early feeding in patients with acute pancreatitis does not increase the risk of adverse events and may reduce hospital length of stay, particularly among individuals with mild to moderate disease [288,289]. However, prolonged fasting in a severe acute pancreatitis model was associated with increased disease severity, including elevated pancreatic enzyme levels, heightened inflammatory cytokines, increased liver oxidative stress, and worsened pancreatic necrosis in mouse model [290]. Conversely, in rat models, fasting has been shown to reduce the severity of cerulein-induced acute pancreatitis, potentially through reduced endogenous cholecystokinin (CCK) release [291]. Fasting, short-term intravenous nutrition, fluid resuscitation, and pain control using narcotics for severe pain or nonsteroidal anti-inflammatory drugs for milder cases are established treatment strategies for acute pancreatitis [292]. However, an American Gastroenterological Association (AGA) Institute Technical Review reported that, despite a large number of observational and randomized trials along with extensive guidelines, systematic reviews, and meta-analyses, many aspects of AP management remain uncertain [293]. Collectively, current evidence highlights the complex relationship between feeding patterns, metabolic health, and pancreatitis outcomes [292,293]. While TRF and TRE show potential metabolic benefits, particularly in reducing obesity-related risk factors, their role in pancreatitis prevention and management remains uncertain [293]. Further preclinical studies are needed to clarify the optimal timing, duration, and application of fasting-based interventions in individuals at risk of or affected by pancreatitis.

## 5. Conclusions

Although pharmacological interventions are effective in the management of prediabetes and type 2 diabetes mellitus, their clinical use may be constrained by adverse effects, underscoring the need for safer and sustainable non-pharmacological approaches. TRF has gained considerable attention as a dietary intervention, with evidence indicating potential benefits for body weight regulation, glycaemic control, insulin sensitivity, and overall metabolic health. Emerging findings also suggest that TRF may influence gut microbiota composition, ameliorate adipose tissue dysfunction and chronic low-grade inflammation, enhance hepatic metabolic function, and limit hepatic steatosis, thereby highlighting its potential as a complementary approach for preventing or delaying the progression of MASH in individuals with type 2 diabetes mellitus.

Despite these promising findings, the current evidence base remains largely preclinical, and several critical knowledge gaps remain. Future investigations should specifically examine the effects of a 14:10 h TRF regimen on glycaemic regulation, gut barrier integrity, microbiota-derived metabolites, systemic and hepatic inflammatory responses, lipid metabolism, and markers of hepatic injury and regeneration using well-characterized animal models of prediabetes. In addition, mechanistic studies are warranted to elucidate how 14:10 h TRF influences the gut–liver axis and inflammatory signalling pathways associated with the progression of prediabetes to type 2 diabetes mellitus and MASH. Long-term investigations are also warranted to evaluate the tolerability, safety, feasibility, sustainability, and durability of the metabolic and hepatic benefits of 14:10 h TRF, and to determine its potential for translation into well-designed human clinical studies. Addressing these research gaps will strengthen the understanding of the mechanisms underlying 14:10 h TRF and establish its clinical feasibility and potential as an evidence-based dietary strategy for preventing or delaying the progression of prediabetes and its associated metabolic complications.

## Figures and Tables

**Figure 1:**
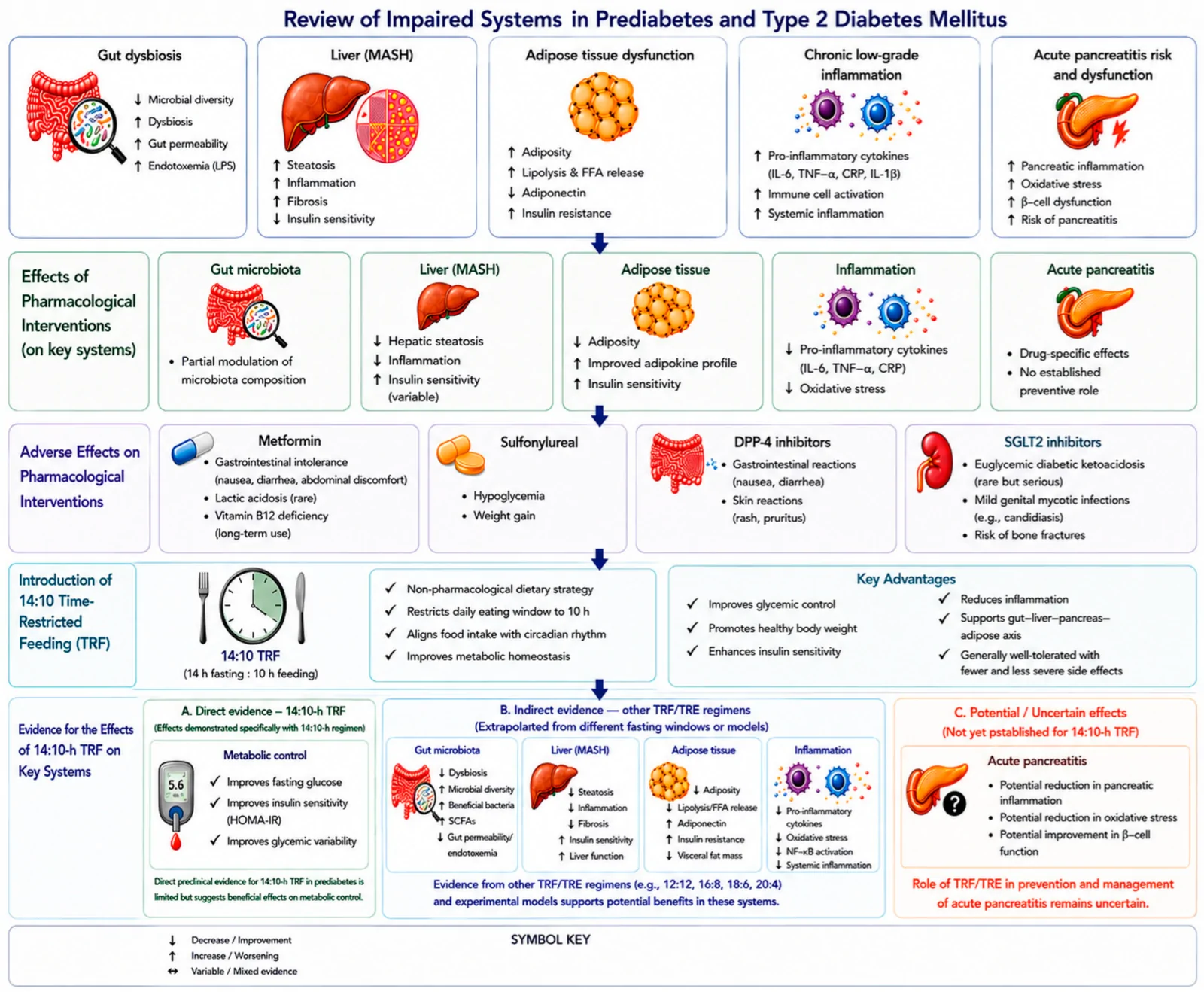
Review of impaired metabolic systems in prediabetes and type 2 diabetes mellitus and the potential effects of 14:10 h TRF. The figure summarizes alterations in gut microbiota, liver (MASH), adipose tissue, chronic low-grade inflammation, and pancreatic function; the effects and adverse effects of commonly used pharmacological interventions; and the proposed benefits of 14:10 TRF. Evidence is distinguished between direct evidence specific to 14:10 TRF, indirect evidence extrapolated from other TRF/TRE regimens, and potential or uncertain effects, particularly in relation to acute pancreatitis. The symbols indicate decreases/improvements, increases/worsening, and variable or mixed evidence.(Msane’s own illustration created using ChatGPT (GPT-5.6 Luna-OpenAI), 2026).

**Figure 2:**
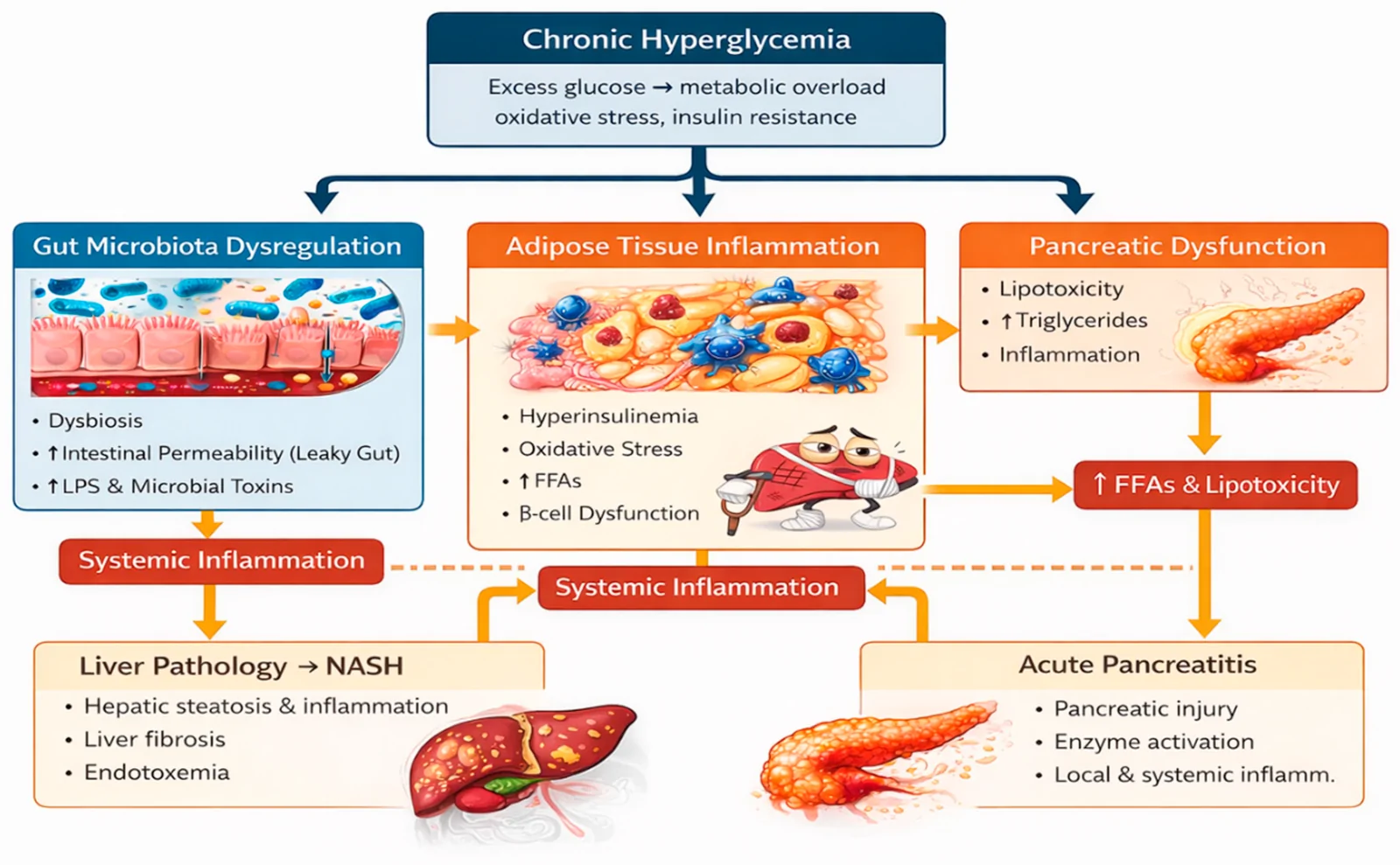
Pathophysiological Impact of Hyperglycemia on Gut Microbiota, Adipose Tissue Inflammation, Acute Pancreatitis, and MASH. (Msane’s own illustration created using ChatGPT (GPT-5.6 Luna-OpenAI), 2026).

**Figure 3:**
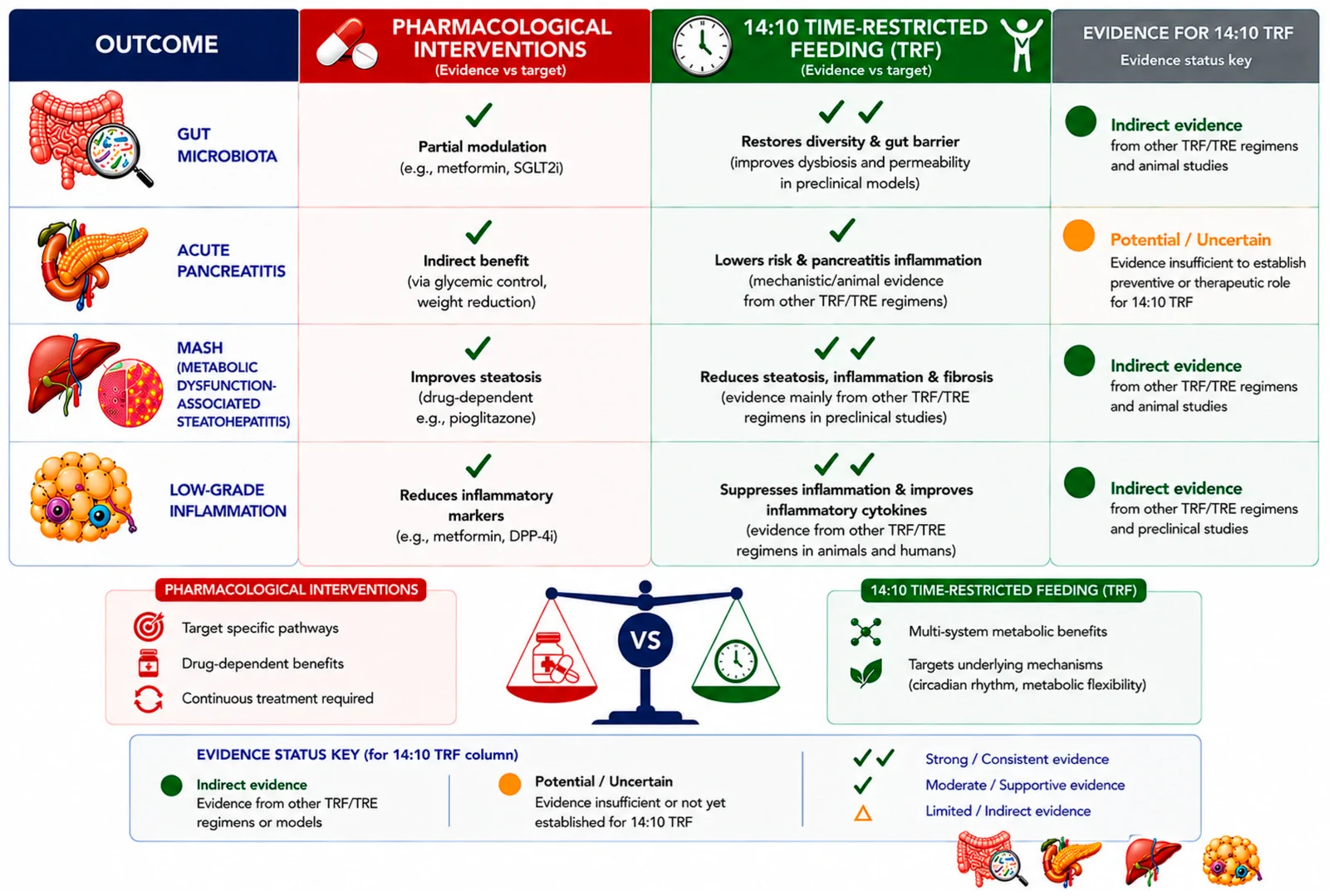
Comparison of pharmacological interventions and 14:10 h TRF across gut microbiota, acute pancreatitis, MASH, and low-grade inflammation. Pharmacological interventions provide target- and drug-dependent effects, whereas 14:10 TRF may influence multiple metabolic pathways, with evidence predominantly indirect or derived from other TRF/TRE regimens. Evidence strength is indicated by check marks and evidence-status indicators. (Msane’s own illustration created using ChatGPT (GPT-5.6 Luna-OpenAI), 2026).

**Table 1:** Summarizes the impact of metformin, sulfonylureas, SGLT2 inhibitors, and DPP-4 inhibitors on gut microbiota, acute pancreatitis, hyperglycemia, adipose tissue function, low-grade inflammation, and MASH. (Reproduced with permission from Chowdhary and others) [138,198,223,233,234,235,243,246,247,248].

Name of the Anti-Diabetic Drug	Evidence Type	Gut Microbiota	Acute Pancreatitis	Adipose Tissue Function	Low-Grade Inflammation	MASH	Adverse Events
Metformin	Randomized clinical trials, observational studies, systematic reviews/meta-analyses and animal studies	Potentially beneficial: Improves gut microbial diversity, promotes SCFAs production, and increases beneficial bacteria	Potentially harmful: Rare cases of acute pancreatitis have been reported, primarily in patients with overdose, renal impairment, or impaired metformin clearance	Beneficial: Improves adipose tissue remodeling	Beneficial: Suppresses chronic inflammation	Beneficial: Improves hepatic steatosis and may reduce the risk of MASH progression	Gastrointestinal intolerance, Lactic acidosis and Vitamin B12 deficiency
Sulfonylurea	Preclinical studies, systematic reviews/meta-analyses, observational studies and clinical studies	Beneficial: Increases beneficial bacteria	Potentially harmful: High risk of acute pancreatitis when compared to other antidiabetic drugs	Potentially beneficial: Improves adipose tissue function by modulating PPARγ signaling	Potentially beneficial: Indirectly reduces low-grade inflammation by improving glycaemic control.	Potential benefit: Reduced risk of MASH	Hypoglycemia and weight gain
DPP4 inhibitor	Randomized clinical trials, observational studies, systematic reviews/meta-analyses, and animal studies	Potentially beneficial: May improve gut microbial composition by reducing dysbiosis.	Uncertain: Large clinical studies suggest no increased risk of acute pancreatitis; however, rare pharmacovigilance reports have raised potential safety concerns.	Potentially beneficial: Improves adipose tissue remodeling.	Potentially beneficial: Reduced low-grade inflammation	Potentially beneficial: May reduce the risk of MASH through hepatoprotective effects	Gastrointestinal and skin reactions
SGLT2 inhibitor	Clinical studies, observational studies, systematic reviews/meta-analyses case reports, and animal studies	Potentially beneficial: Improves gut microbial diversity and increases beneficial bacteria	Potentially harmful: Rarely associated with acute pancreatitis; evidence for an increased risk remains inconsistent.	Potentially beneficial: Improves adipocyte function and metabolic regulation.	Potentially beneficial: Reduces chronic low-grade inflammation.	Beneficial: Improves hepatic steatosis, insulin resistance, and liver function, and may reduce the risk of MASH progression	Euglycemic diabetic ketoacidosis, Mild genital mycotic infections and Risk of bone fractures

## Data Availability

No new data were created or analyzed in this study. Data sharing is not applicable to this article.

## Abbreviations

ACE	Angiotensin-converting enzyme
ADA	American Diabetes Association
AGA	American Gastroenterological Association
ALA	Alpha-lipoic acid
AL	ad libitum
ALT	Alanine aminotransferase
AST	Aspartate aminotransferase
ALP	Alkaline phosphatase
AMLN	Amylin liver NASH
AMPK	Adenosine monophosphate-activated protein kinase
AP	Acute pancreatitis
ARB	Angiotensin receptor blockers
BMI	Body mass index
CBDL	Common bile duct ligation
CCK	Cholecystokinin
CD	Crohn’s disease
CFTR	Cystic fibrosis transmembrane conductance regulator
CTRC	Cationic trypsinogen, chymotrypsin C
CVD	Cardiovascular disease
DASH	DPP-4 activity and/or structure homolog
DPP	Dipeptidyl peptidase
DI GM	Dietary Index for Gut Microbiota
eTRE	Early time-restricted eating
GI	Gastrointestinal
GLUT	Glucose transporter
HF	High fat
HFCD	High-fat, high-carbohydrate diet
IBD	Inflammatory bowel diseases
IFG	Impaired fasting glucose
IGT	Impaired glucose tolerance
IHTG	Intrahepatic triglycerides
IL	Interleukin
LCD	Low-calorie diet
lTRE	Late time-restricted eating
LPSs	Lipopolysaccharides
M	Macrophage
MAP	Mild acute pancreatitis
MASH	Metabolic dysfunction-associated steatohepatitis
MASLD	Metabolic Dysfunction-Associated Steatotic Liver Disease
MKC	Mono-keto-cholic
MODS	Multiple organ dysfunction syndrome
MTZ	Metronidazole
NAFLD	Non-alcoholic fatty liver disease
NASH	Non-alcoholic steatohepatitis
NSAID	Non-steroidal anti-inflammatory drugs
NHANES	National Health and Nutrition Examination Survey.
Ox-mtDNA	Oxidized mitochondrial DNA
PDAC	Pancreatic ductal adenocarcinoma
PRSS1	Protease serine 1/cationic trypsinogen
SAP	Severe acute pancreatitis
SCFA	Short-chain fatty acid
SGLT	Sodium–glucose transporters
SIRS	Systemic inflammatory response syndrome
SPINK1	Serine protease inhibitor Kazal type 1
TMAO	Trimethylamine-N-oxide
TRF	Time-restricted feeding
UCP1	Uncoupling protein 1
VAT	Visceral adipose tissue
VLDL	Very low-density lipoprotein
WAT	White adipose tissue
WHO	World Health Organisation
